# The risk of pulmonary tuberculosis in underground copper miners in Zambia exposed to respirable silica: a cross-sectional study

**DOI:** 10.1186/s12889-016-3547-2

**Published:** 2016-08-23

**Authors:** Kingsley Ngosa, Rajen N. Naidoo

**Affiliations:** 1 Occupational Health and Safety Institute, P.O. Box 20205, Kitwe, Zambia; 2 Discipline of Occupational and Environmental Health, University of KwaZulu-Natal, Durban, 4041 South Africa

**Keywords:** Pulmonary Tuberculosis, Silica dust, Underground copper miners, Cumulative exposure, Smoking

## Abstract

**Background:**

Pulmonary tuberculosis (PTB) among underground miners exposed to silica remains a global problem. Although well described in gold and coal mining, risk in other mining entities are not as well documented. This study aims to determine dust-related dose response risk for PTB among underground miners exposed to silica dust in Zambia's copper mines.

**Methods:**

A cross sectional study of in-service miners (*n* = 357) was conducted at Occupational Health and Safety Institute (OHSI), Zambia. A systematic review of medical data over a 5-year period from assessments conducted by doctors at OHSI and statutory silica exposure data (*n* = 16678) from the Mine Safety Department (MSD) were analysed. Lifetime cumulative exposure metrics were calculated. Multivariate logistic regression analysis was used to determine the association between PTB and lifetime exposure to silica, while adjusting for various confounders.

**Results:**

The median respirable silica dust level was 0.3 mg/m^3^ (range 0.1–1.3). The overall prevalence of PTB was 9.5 % (*n* = 34). High cumulative respirable silica dust category showed a statistically significant association with PTB (OR = 6.4 (95 % CI 1. 8–23)) and a significant trend of increasing disease prevalence with increasing cumulative respirable silica dust categories was observed (p_trend_ < 0.01). Smoking showed a statistically significant association with PTB with OR = 4.3 (95 % CI 1.9–9.9).

**Conclusions:**

Our results demonstrate the association of increased risk for certified active TB with cumulative respirable dust in a dose related manner among this sample of copper miners. There is need to intensify dust control measures and incorporate anti-smoking interventions into TB prevention and control programmes in the mines.

## Background

Pulmonary Tuberculosis (PTB) is a serious public health problem facing mineworkers, not only in Zambia but in most developing countries. The tuberculosis (TB) incidence in miners in Southern Africa is reportedly the highest among any working population [1].

Silica is a major component of sand and rocks and workers at risk of exposure, apart from the mineral ore miners, are tunnel drillers and those involved in quarry work, foundries, and excavation [2].

Mining activities expose workers to silica dust, which has contributed substantially to the TB epidemics in southern Africa and other low income regions of the world [3]. About 1.7 million workers in the United States are potentially exposed to silica dust [4]. Several studies among South Africa goldminers, particularly in studies of ex-miners, have found high prevalences of PTB, ranging from 23.9 to 35 % [5–8] and increased mortality rate from TB among white South Africa gold miners followed up for 20 years compared with the general white population [9]. Some researchers have noted increased TB prevalence with duration of exposure. Increased duration and levels of exposure have been associated with increased risk among goldminers [10], and crystalline silica exposed industrial workers in USA [11]. This risk may persist after exposure ceases [12]. Despite lower prevalence among South African coal miners (5.2 %), increased risks for TB have also been described [13].

There is limited reporting in the literature of PTB among copper miners. One study described a high prevalence of silicosis (35 %) in former Brazilian copper miners with 4 to 40 years of exposure, and with 11 % of the miners having TB, emphysema and COPD [14].

This study was aimed at determining the dust-related dose response risk for PTB among mineworkers exposed to silica dust in the copper mines in Zambia.

## Methods

### Study design and setting

This cross-sectional study was conducted for in-service miners examined between 2005 and 2010 at the Occupational Health and Safety Institute (OHSI), a statutory agency in Kitwe, Zambia where miners report for annual mandatory medical examinations. Any person aged between 18 and 59 years, working or seeking to work in the mine, is subjected to an annual mandatory medical examination. Prospective miners are brought to the institution by companies intending to employ them, while others present themselves for initial medical examinations. Each miner, on first presentation to the Institute (initial medical examinations), is assigned a unique Institute number which becomes his/her personal number for as long as he/she remains working in the mining industry.

The medical examinations consist of medical and occupational history, clinical examinations, laboratory investigations and chest radiographs on all prospective miners. Sputum for acid fast bacilli (AFB) and erythrocyte sedimentation rate (ESR) are done on miners with abnormal chest radiographs (CXRs). Additional investigations such as spirometry and audiometry are done depending on the findings from the initial assessment. Prospective miners with a history of TB or radiological scarring suggestive of past TB are not permitted to work underground and are not employed in the mines. At each mandatory annual medical assessment, a 12-month fitness-to-work certificate is issued, obliging workers to return the following year, if continued employment is required. For miners certified with pneumoconiosis or work-related cardiorespiratory TB, additional information such as date of disease certification, age at certification and duration of employment before certification, is entered into the Institute medical files. A miner who has been certified as having PTB is relocated to non-dusty areas of the mine or discharged. A miner found to have PTB can only be certified if he has worked in the scheduled area for more than 12 completed months. PTB, which has been diagnosed in a miner with a work history of less than 12 months in an area with a risk of exposure to silica dust is considered non work-related and is not certified. All work-related diagnosed PTB is certified for compensation purposes. All the information on miners is entered and stored safely onto the OHSI medical files.

The study involved the review of miners’ medical records maintained at the OHSI. The medical records contained information on health status of the miner and CXR report read from posteroanterior (PA) view of the standard-size chest radiographs. These were read by the medical panel at the time the miner was being screened. The process of screening and examinations at the Institute involves medical and occupational history taking, and clinical examination of miners done by individual medical officers. All the information gathered by the medical officers is forwarded to the medical panel which sits on daily basis. The medical panel is mandated to read all the CXRs with laboratory findings and award medical certificates. The medical panel consists of the screening medical doctors together with medical doctors with B-reader training and one an occupational medicine specialist with substantial experience in reading CXRs, including distinguishing between radiological active and old PTB. Non-TB lesions on CXRs were distinguished from TB by the medical panel after reviewing both the medical and occupational history, clinical findings and radiological findings such as cavitations, military mottling, hilar adenopathy and the presence of opacities in the upper and middle lobes which are more suggestive of TB. The final decision of the medical panel was made by consensus. For the purposes of this study, the outcome used was certified TB, which was defined as sputum smear positive or CXR for active TB, meeting the criteria for work-relatedness as described above. The CXR findings which were reported by the medical panel at the time of examination and was recorded in the file was used in this research. CXRs were not subjected to re-reading. All non-TB abnormalities were aggregated during coding, making it impossible to retrieve data on silicosis specifically.

### Population and sample selection

Included in the study, were seven major copper mines with underground operations. The newly opened mines and open pit mines were excluded from the study. The participating mines where chosen because they had a long history of underground mining, a large number of employees (more than a 1000 miners) and no history of mine closures. Three mines met the inclusion criteria and accepted invitation to participate. All in-service miners examined at OHSI between 1 January 2005 and 31 December 2010 from the participating mines and who had worked for 12 completed months or more, were eligible for selection as study subjects (*N* = 5840). Exclusion of ex-miners was necessary as the exposure data for periods of their past employment (1960’s and 1970’s) was absent. The unique Institute identification numbers was used as sampling frame. The study sample was selected using a 1 in 16 systematic random sampling method, to provide a total sample of 360, which was estimated as being appropriate to determine whether an association between exposure and PTB truly existed among the sample. The medical files were used as the sampling frame and captured through the Institute numbers.

### Exposure assessment

The exposure data from the Mine Safety Department (MSD), the government agency responsible for the monitoring of the mining environment was obtained for the mines under study. The data (*n* = 16678) was in the form of dust counts from various mine worksites such as loading bay and crushers and processes like lashing and drilling, using the Rand Konimeter for ‘snap’ sampling. It was taken by occupational hygienists and qualified technicians from the ventilation departments of respective mines. Random checks and sampling of dust were carried out by the MSD to monitor compliance and accuracy of the results they received from the mines. The reports contained information on mine name, date of collection, major worksites (surface or underground), sections and processes. These legally mandatory reports are presented on quarterly basis to the MSD. Legislation demands that all work sites are periodically sampled at least every 90 days. Dust reports from 1990 to 2010 were available for the research. The silica dust levels in the work sites and processes were converted to equivalent gravimetric silica estimates by applying the conversion factor of 0.09 mg/m^3^ to convert from million particles per cubic foot (mppcf) to mg/m^3^ (i.e 1ppcc = 0.003 mg/m3) suggested by previous researchers [15, 16]. These levels were then allocated to each selected miner based on the job title and the area the miner had been operating from within a particular mine in a specified time, using the occupational history from the medical records. The dust levels were allocated for each of these strata, and summed across the entire lifetime of employment in the industry to obtain cumulative dust exposures (CDE), according to the formula:

$$ CD{E}_i={\displaystyle \sum_{jk}{M}_{jk}{Y}_{ijk}} $$

where *M*_*jk*_ is the mean dust level for work title, *j* in work area, k and *Y*_*ijk*_ is the time in years spent by subject *i* in work title, *j* and area *k*.

No participant was employed in the period prior to 1990.

### Statistical analysis

The data for in-service miners collected were coded and captured in Epidata version 3.1 (EpiData Association, Odense, Denmark). Statistical analysis was conducted using Stata IC version 13.1 software for Windows (StataCorp LP, College Station, TX). Means and standard deviations (SD) were compared for numerical variables using student’s *t*-test. Mann-Whitney *U* test was used to compare medians for data not normally distributed. Pearson’s chi-square test was used to test for association with PTB for categorical variables. Analysis of variance (ANOVA) test was used to compare means across the three mines.

Cumulative respirable silica dust exposure (CDE) was calculated by the product of annual mean concentration of respirable silica dust of the section worked and years worked in that section, summed for all sections, over the lifetime employment in the copper mining industry. It was calculated from the start of mining to the date of certification of PTB, or end of the study period (2010) for non-PTB cases. For analysis, CDE was divided into tertiles: low (cumulative respirable silica dust below 1.0 mg.yrs/m^3^), medium (cumulative respirable silica dust from 1.01 to 1.82 mg.yrs/m^3^) and high (cumulative respirable silica dust more than 1.82 mg.yrs/m^3^). Exposure categories were also used to analyse trends of disease prevalence with increasing cumulative respirable silica dust by comparing the prevalence of certified active TB in the medium and high cumulative respirable silica dust category with low cumulative respirable silica dust category as a reference category.

Multivariate logistic regression was used to determine the association between certified active TB, and cumulative dust exposure categories, adjusted for age at certification, gender and smoking status. In a separate model, CDE was also run as a continuous variable to determine the association with certified active TB, adjusting for age at certification, gender and smoking status. Miners with unknown smoking status (*n* = 11) were not included in the model. Odds ratios with 95 % confidence intervals were calculated and presented. All analyses were conducted with a significance level of 5 %.

## Results

Of the 360 files of miners in the original random sample selected from the mines, 3 (0.8 %) samples comprised of females. Because of the few number of females, they were excluded from analysis. There were no significant differences in the demographics across the mines. Altogether, 51 (14.3 %) of the miners were smokers at the time of employment. There was no data on smoking for 11 (3.1 %) (Table 1). Of the 34 miners diagnosed with PTB, 16 (47.1 %) and 18 (52.9 %) were sputum positive and negative on direct microscopy for AFB respectively.

**Table 1:** Demographic factors and clinical findings of underground copper miners from selected mines in Zambia

Characteristics	Mine 1 (*n* = 137)	Mine 2 (*n* = 99)	Mine 3 (*n* = 121)
Age at certification^a^, years, (mean (SD)) (Range)	30.8 (5.9) (19.6–49.1)	31.9 (6.9) (21.2–54.2)	31.3 (6.0) (19–52.2)
Sex, n, (%)			
Male	137 (100)	99 (100)	121 (100)
Smoking, n, (%) ^b^			
Non-smokers	121 (88.3)	76 (76.8)	98 (81)
Smokers	14 (10.2)	17 (17.2)	20 (16.5)
Unknown	2 (1.5)	6 (6.1)	3 (2.5)
Chest radiographs, n, (%)			
Normal	117 (85.4)	82 (82.8)	98 (81)
Abnormal (PTB) ^c^	12 (8.8)	7 (7.1)	14 (11.6)
Abnormal (Non-PTB) ^d^	8 (5.8)	10 (10.1)	9 (7.4)
AFB Positive n, (%)	4 (33.3)	5 (62.5)	7 (50)
ESR mm/h, mean, (SD) (Range)	76.9 (31.0) (35–127)	73.3 (25.2) (30–108)	85.2 (22.1) (54–133)

*ESR* Erythrocyte sedimentation rate (positive if >20 mm/h)

*AFB* Acid-fast bacillus

^a^Age at certification – mean age of the miners at the time of certification compared across the Mines

^b^Smoking status –current smokers at employment

^c^Abnormal radiographs (PTB) = Chest radiographs suggestive of PTB

^d^Abnormal radiographs (Non PTB) = Chest radiographs suggestive of chest pathology other than PTB

There were no statistical significant differences across the mines with respect to clinical characteristics (Table 1), duration of service or silica dust exposure (Table 2). The duration of exposure was short, with the mean years of exposure of 5.0 (Mine 2) and 5.2 (Mines 1 and 3) (Table 2). Median exposure levels for both overall mine and for specific job descriptions, exceeded the newly revised Occupational Safety and Health Administration Permissible exposure level (OSHA-PEL) of 0.05 mg/m^3^ (Table 2). In addition, more than two-thirds of all sampling points (*n* = 16678) were also above this standard. With the exception of the high levels recording for crushing and blasting tasks at Mine 1, the median exposure levels for other job descriptions were in a narrow range from 0.2 to 0.4 mg/m^3^.

**Table 2:** Exposure characteristics of miners (*n* = 357)

Characteristics	Mine 1 (*n* = 137)	Mine 2 (*n* = 99)	Mine 3 (*n* = 121)
Length of service, years, Mean (SD) (Range)	5.2 (2.7) (1.0–13.1)	5.0 (2.5) (1.1–11.4)	5.2 (2.3) (1.2–11.0)
Overall respirable silica dust mg/m^3^, Median (Range)	0.4 (0.0–1.0)	0.2 (0.1–1.3)	0.2 (0.1–0.5)
Silica dust in sections mg/m^3^, Median (Range)			
Crusher Attend	0.6 (0.1–1.0)	0.3 (0.1–0.9)	0.3 (0.1–0.5)
Blasting Tech/Foreman	0.5 (0.2–0.9)	0.4 (0.1–1.1)	0.3 (0.1–0.4)
Lasher	0.4 (0.0–1.0)	0.2 (0.1–1.0)	0.2 (0.1–0.4)
Driller	0.4 (0.2–0.9)	0.2 (0.1–0.9)	0.2 (0.1–0.5)
Trammer	0.4 (0.2–0.8)	0.3 (0.1–1.3)	0.2 (0.1–0.4)
W/shop foreman/Attend	0.4 (0.1–0.7)	0.2 (0.1–0.6)	0.2 (0.1–0.4)
Others-workman	0.4 (0.1–0.9)	0.2 (0.1–1.0)	0.2 (0.1–0.4)
Cumulative respirable silica dust mg.y/m^3^, Mean (SD) (Range)	1.3 (0.7) (0.3–3.2)	1.1 (0.5) (0.3–2.2)	2.3 (1.0) (0.7–4.7)

*SD* Standard deviation, *tech* Technician, *Attend* attendant, *W/shop* workshop

Medium and high CDE categories, age at certification, smoking status, and length of service were significantly associated with PTB (Table 3). There was a statistically significant increasing trend (p_trend_ < 0.01) of disease prevalence with increasing categories of dust exposure (low CDE = 2.5 % (3); medium CDE = 8.7 % (10) and high CDE = 17.1 % (21)).

**Table 3:** PTB status across the variables of interest

Characteristics	PTB cases (*n* = 34)	No PTB (*n* = 323)
Age at certification, years, Mean, (SD) ^a*^	34 (6.0)	31.0 (6.2)
Smoking status^b^		
Non-smokers, n, (%)	22 (64.7)	273 (84.5)
Current smokers, n, (%)^**^	12 (35.3)	39 (12.1)
Length of service, Mean, (SD) ^a*^	6.1 (2.0)	5.1 (2.5)
Sex		
Male, n, (%)	34 (9.5)	323 (90.5)
Cumulative respirable silica Dust Exposure, Mean, (SD) ^a**^	2.2 (1.0)	1.5 (0.9)
Cumulative respirable silica dust categories^b^		
Low, n, (%)	3 (8.8)	116 (35.9)
Medium, n, (%)^*^	10 (29.4)	105 (32.5)
High, n, (%)^**^	21 (61.8)	102 (31.6)

*p-value < 0.05; ** *p*-value < 0.01

^a^t-test

^b^Chi-square test

The high CDE category, compared with the lowest category, was statistically significantly associated with PTB (OR = 6.4 (95 % CI 1.8–23.0)), after adjusting for age, gender and smoking status. Medium CDE showed a non-statistically significant elevated risk but lower than that of the highest category, suggesting a dose-related gradient. Current smoking, as compared to a current non-smoker also presented as a statistically increased risk for the development of PTB (Table 4).

**Table 4:** Multivariate logistic regression analyses of independent factors as predictors of PTB

Variables	OR	95 % CI
Age at certification	1.1	1.0–1.1
Current smoker*	4.3	1.9–9.9
Cumulative respirable silica dust exposure		
Low	1	
Medium	2.8	0.7–10.6
High**	6.4	1.8–23.0

Miners with unknown smoking status not included

Current smokers compared with current non-smokers

*CI* confidence interval, *OR* odds ratio

*p* < 0.01*, *p* < 0.05**

A model which included cumulative respirable silica dust exposure as a continuous variable was also run, adjusting for age, sex and smoking. A definite dose response was seen: for each unit increase in silica levels for each year of exposure, there was almost a twofold increase in PTB risk (OR: 1.9; 95 % CI: 1.3–2.8).

## Discussion

In this record review of 360 Zambian underground copper miners, certified active TB was associated with increasing cumulative silica exposure, despite the relatively short duration of exposures on these selected mines. To the best of our knowledge, this is the first such description among copper miners.

Our study established a prevalence of certified active TB in underground miners in Zambia of 9.5 %. The higher prevalences of TB found in the gold mines than in the present study in copper mines could probably be due to the differences in the definition of TB used across the studies, and in the varying silica concentration between the gold and copper mines. In the Zambian copper mines, the silica content in the atmospheric dust was reported to be in the range of 19 to 43 % [17] with respirable silica dust levels established in this study of 0.1–1.3 mg/m^3^, but in the gold mines, the reported routine respirable dust measurements are in the range of 0.02–4.29 mg/m^3^ with mean silica fraction of 16 % [18]. Other researchers have reported silica concentrations in the gold mines as high as 54 % after acid washing and incineration [19]. The reported dust levels in the coal mines in South Africa are in the range of 0.9–2 mg/m^3^ with silica content reportedly to be below the regulatory action level of 5 % [20], with the prevalence of TB on these mines ranging from 3.6 to 5.4 % [13]. Dust concentrations across studies may vary depending on the source, treatment and methods of analysis and these differences must be cautiously interpreted.

It is likely that the differences found in our study could also be related to the age of the miners (mean age = 31.3 years). Additionally, the duration of exposure could provide another explanation. In the current study, the miners had a short mean working history of about 5 years and a relatively younger age group and could explain why there was no association between age at certification and PTB after adjusting for smoking, gender and cumulative respirable silica dust categories, although by bivariate analysis, association was significant.

Silica exposure has been well documented to be associated with PTB [5, 8, 9, 11, 12, 21, 22]. While these findings have been shown in a variety of mineral settings, such as gold [5–8, 10, 12], coal [13], foundry workers [23] and quartz stone crushers [24], this has not been shown previously within copper mines, where levels of silica are not typically as high as in other settings. The risk of PTB in the copper mines is not well established as compared with the gold mines. Our data demonstrated increased risk for certified active TB with increased exposure to silica dust.

From the respirable silica dust established by this study, the estimated median quartz levels in the dust on the mines under study ranged from as low as 0.01 mg/m^3^ for the mine with the lowest silica content of 19 % to as high as 0.6 mg/m^3^ for the mine with the highest silica content of 43 %. More than two- thirds of the dust data points were above the now revised OSHA-PEL of 0.05 mg/m^3^. Before the OSHA-PEL was revised downwards to 0.05 mg/m^3^, mines were using 0.1 mg/m^3^ as Occupational Exposure Limit (OEL).

Miners were at risk of developing silicosis when exposed to levels at or below 0.1 mg/m^3^ [5, 18, 25, 26]. Thus, it is unlikely that this level is protective against PTB – as is indicated by our data.

In addition to respirable silica exposure, we found a strong association between smoking and certified active TB. This supports population based studies where smoking has been shown to increase the risk of PTB [27–32]. The risk estimates in these studies range from 1.61 to 4.5, similar to those found in our study. The comparison with non-mining study populations must be viewed with caution, given the differences in case definitions.

The strengths of our study were the ability to recreate exposure profiles from a sample of miners from time of employment until certification of disease. This detailed occupational history and exposure data was used to estimate cumulative exposures for each miner for all the years worked as opposed to current exposures and length of service being used as a proxy to exposure to estimate cumulative exposures. This minimized exposure misclassification. The exposure data were collected by qualified technicians and hygienists. In addition, the clinical evaluation conducted by a group of trained and experienced physicians at OHSI provided us with access to quality data.

The other merit of this study is that it is the first study to look at the relationship between PTB and exposure to silica dust among underground mineworkers in copper mines in Zambia or elsewhere, as well as the dose response impact of dust exposure on frequency of pulmonary TB.

The study limitations included lack of data on other risk factors for PTB, such as previous TB, TB contacts, socioeconomic factors, Human Immunodeficiency Virus (HIV) status and past smoking status which could have influenced the relationship between PTB and exposure. Absence of cultures for TB is another study limitation with a potential of TB misclassification and an effect on the accuracy of active TB prevalence. Past history of TB is likely to have been minimal, as this would have been detected on the initial medical assessment, which includes a worker interview, clinical assessment and chest radiograph, and those with such history or radiological scarring suggestive of past TB are not permitted to work underground or in scheduled areas and were not part of this study. Other factors, such as past smoking status, TB contact, overcrowding and HIV status is unlikely to have had a differential distribution across the exposure categories, and thus only minimally likely to have influenced the estimates of risk. The estimated prevalence of HIV in Zambia among the 15–49 years age group is 12.5 % [33]. Furthermore, the prevalence of TB in 2012 in Zambia was 388 per 100,000 population [34], which is lower than that established by this study (9.5 %). Lack of information on the use of personal protective equipment (e.g masks) is another limitation factor but is likely to also have been non-differentially distributed across the sample. The lack of information on silicosis made it impossible to determine the relationship between TB, respirable silica and silicosis in this sample.

The other study limitation is the exposure data. Konimeter measurements which are based on optical counting of the number of particles in a portion of sample are prone to analytical variability arising from collection and analytical methods. It is least reliable for high dust count as opposed to low dust count [35] and could have affected this study.

## Conclusion

The findings of this study add to the existing evidence that occupational exposure to silica dust poses a high risk of TB in underground miners, even in conditions of short duration of dust exposure. This study underlines the need to maintain the dust levels below international standards to lower the prevalence of TB further through dust control.

## Abbreviations

AFB: Acid fast bacilli
CDE: Cumulative silica dust exposure
COPD: Chronic obstructive pulmonary disease
CXR: Chest x-ray or radiograph
ESR: Erythrocyte sedimentation rate
HIV: Human Immunodeficiency Virus
MSD: Mine safety department
OHSI: Occupational Health and Safety Institute
PA: Posteroanterior
PTB: Pulmonary tuberculosis
TB: Tuberculosis
